# The Efficacy of Telehealth-Delivered Speech and Language Intervention for Primary School-Age Children: A Systematic Review

**DOI:** 10.5195/ijt.2017.6219

**Published:** 2017-06-29

**Authors:** DANIELLE WALES, LEISA SKINNER, MELANIE HAYMAN

**Affiliations:** SCHOOL OF HEALTH, MEDICAL AND APPLIED SCIENCES, CENTRAL QUEENSLAND UNIVERSITY, ROCKHAMPTON QLD, AUSTRALIA

**Keywords:** Intervention, Language, Primary School-age, Service Delivery, Speech, Speech-language Pathology, Telehealth

## Abstract

The purpose of this article is to determine if telehealth-delivered speech-language pathology interventions are as effective as traditional in-person delivery for primary school-age children with speech and/or language difficulties. A systematic review was conducted (in accordance with PRISMA guidelines) using five databases, two journals and reference lists. Titles and abstracts were screened for inclusion, with relevant studies reviewed in full-text. Initial searches identified 132 articles. Following exclusion of non-relevant studies, seven articles remained for inclusion. Results revealed both telehealth and in-person participants made significant and similar improvements when treatment effects were measured through five of the six outcome measures. Findings showed there is limited but promising evidence to support telehealth for delivering speech-language pathology intervention services to school-age children. Whilst this is encouraging, particularly for rural children where in-person services are limited, more rigorous study designs are required to support the efficacy of telehealth for this population.

In Australia, over 17% of children are considered to be vulnerable (<10th percentile) or at-risk (10–25th percentile) for developing impairments in language and literacy (McCormack & Verdon, 2015). Another 25% of children are vulnerable or at-risk for developing impaired communication skills (McCormack & Verdon, 2015). The majority of these children are located in rural communities across Australia (McCormack & Verdon, 2015). Despite the high proportion of vulnerable/at-risk children residing within these communities, access to specialist care services, such as speech-language pathology (SLP), is limited. In fact, recent data (Health Workforce Australia [HWA], 2014) suggests that less than 24% of all employed speech-language pathologists in Australia work within these regions, indicating that a significant imbalance exists between the SLP services available in rural areas compared to major cities. Specifically, research suggests that between 0.59 and 1.69 speech-language pathologists are available per 10,000 people in very remote and outer regional areas of Australia, compared to 2.59 speech-language pathologists per 10,000 people in the major cities (HWA, 2014).

Regardless of location, speech and language skills are a strong predictor of success in education, social participation, and employment. Children with communication difficulties progress more slowly in reading and writing and experience increased bullying and poorer peer relationships (Law, Boyle, Harris, Harkness, & Nye, 1998; McCormack, Harrison, McLeod, & McAllister, 2011). Speech and language difficulties not only threaten academic performance during the school years, but also have a considerable impact on social and vocational inequalities in adulthood (Johnson, Beitchman, & Brownlie, 2010; Law et al., 1998; Ruben, 2000; Schoon, Parsons, Rush, & Law, 2010; Whitehouse, Watt, Line, & Bishop, 2009). In particular, individuals with persisting communication problems have increased difficulty interacting with others, sustaining employment, and living independently (Clegg, Hollis, Mawhood, & Rutter, 2005). A history of communication difficulties is also linked to a higher rate of psychiatric disorders, particularly anxiety (Beitchman et al., 2001). Given the prevalence and associated impact of communication difficulties in rural Australian children, it is important that SLP intervention is available to these children to assist in the development of vital communication skills (Law, Garrett, & Nye, 2003).

As a solution to the inequity of access to SLP services in rural areas, some practices/practitioners have begun to make use of an innovative service delivery approach, commonly referred to as telehealth (Speech Pathology Australia [SPA], 2014). This term refers to “the application of telecommunications technology to deliver clinical services at a distance by linking clinician to client, caregiver, or any person(s) responsible for delivering care to the client, for the purposes of assessment, intervention, consultation and/or supervision” (SPA, 2014, p. 4). The governing body of the SLP profession, Speech Pathology Australia (SPA), supports and encourages the use of telehealth but recommends that the services provided through this service delivery model be “equivalent to standard clinical care” (SPA, 2014).

Telehealth-delivered SLP services have previously been investigated in reviews regarding a number of practice areas and populations. Mashima and Doarn (2008) conducted an extensive literature review on the application of telehealth in SLP with adults and a small number of studies with children. They reviewed 40 studies investigating disorders relating to adult neurogenic communication, fluency, voice, dysphagia (n=35), and childhood speech and language (n=5). This review suggested that telehealth is a feasible and effective method for providing SLP services at a distance. However, the authors noted that the reviewed literature consisted primarily of pilot studies and anecdotal accounts of telehealth applications rather than large, well-controlled, randomised clinical trials (Mashima & Doarn, 2008). Reynolds, Vick, and Haak (2009) conducted a narrative review of 29 studies which were analysed using a quality assessment checklist. These 29 articles focused on assessment and intervention with the adult (n=19) and paediatric (n=7) population as well as an unspecified population (n=3). The authors concluded that the results achieved through the telehealth and in-person service delivery models were equivalent; however, many of the studies noted that telehealth was not a complete replacement for in-person services but may be appropriate for combined practices. These findings were consistent with the review conducted by Theodoros (2012), which investigated 19 studies regarding adult neurogenic communication, voice, stuttering, dysphagia and laryngectomy follow-up and four studies regarding paediatric speech, language and literacy disorders. Edwards, Stredler-Brown, and Houston (2012) conducted a further review investigating 39 studies in the fields of audiology and SLP. The majority of these studies were conducted on adult populations (n=27) with neurogenic communication, voice, dysphagia and fluency disorders. The review was further expanded to include a small number of studies (n=12) focusing on early intervention services. This review by Edwards et al. (2012) suggested that telehealth is an effective way to diagnose and treat both adults and children in the areas investigated, as services provided through telehealth or by conventional in-person means resulted in similar outcomes.

Although these previously conducted reviews included studies targeting the paediatric population, the number of studies investigated was minimal and the focus was primarily on the application of telehealth in SLP with the adult population. These reviews suggest positive results. However, service delivery models and intervention techniques used with children typically differ from those used with adults, as the focus with children tends to be on achieving developmental milestones, as opposed to a rehabilitative approach that is commonly used with adults (Edwards et al., 2012). It can therefore be difficult to apply previous findings that were obtained from primarily adult-focussed studies to the paediatric population.

The potentially detrimental effects of communication difficulties on a child’s education and social participation increase the importance of alleviating these where possible, regardless of where the child resides. It is therefore important to focus on this specific population to determine whether telehealth service delivery may be a viable alternative to in-person intervention in locations where this service is not readily available. However, no review to date has focused specifically on evaluating the telehealth studies undertaken with children. Thus, this systematic review evaluated the present literature to determine if telehealth-delivered SLP interventions are as effective as traditional in-person delivery for primary school-age children with speech and/or language difficulties.

## METHODS

**Figure 1:**
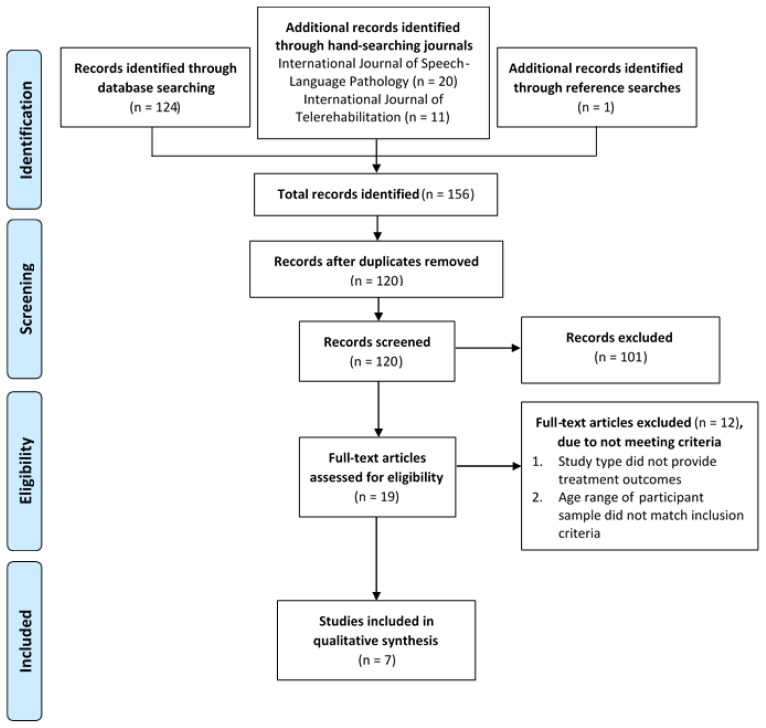
PRISMA Flow Chart showing search and selection process that yielded the final seven articles (adapted from Preferred Reporting Items for Systematic reviews and Meta-Analyses [PRISMA]; Liberati et al., 2009). *Note.* From Moher D, Liberati A, Tetzlaff J, Altman DG, The PRISMA Group (2009) Preferred Reporting Items for Systematic Reviews and Meta-Analyses: The PRISMA Statement. PLoS Med 6(7): e1000097. https://doi.org/10.1371/journal.pmed.1000097

To address this study’s aim, a systematic review was conducted in accordance with the Preferred Reporting Items for Systematic reviews and Meta-Analyses (PRISMA) guidelines (Liberati et al., 2009). The PRISMA flow chart is detailed in Figure 1 (adapted from Liberati et al. (2009)). The current systematic review was registered with the PROSPERO registry: CRD42016052187.

### SEARCH STRATEGY

A systematic literature search was undertaken using the PubMed, CINAHL, Scopus, ERIC and SpeechBITE databases. Additional manual searches in two highly relevant journals, the International Journal of Speech-Language Pathology and the International Journal of Telerehabilitation, were also conducted, in order to locate more recent versions of journals that may not yet have been transferred into the databases. Systematic search strategies were adhered to using the following search string: (telehealth OR telepractice OR telerehabilitation OR teletherapy) AND (speech pathology OR speech-language pathology OR speech therapy) AND (child OR paediatric). In addition, citations and references within identified articles were searched for further studies relevant to the review. The authors corresponded with experts in the field to ensure all relevant studies were included within the review.

### STUDY SELECTION

The studies identified through the systematic searches were included in the review if they reported studies of speech and language intervention delivered through telehealth to primary school-age children (4–12 years) across various settings (e.g., schools, private practice), provided treatment outcome data on intervention effectiveness and did not describe special client populations (e.g., autism spectrum disorder, childhood apraxia of speech). The year of publication was not restricted, ensuring all available evidence was identified, but the search was limited to articles written in English. Papers were included on speech intervention (speech sound production and intelligibility) and language intervention (receptive and expressive language). Articles describing voice, fluency, pragmatics, literacy or special client populations were excluded to focus on primary speech and language disorders.

### DATA EXTRACTION

All articles identified from the initial searches were reviewed and duplicates were removed. The title and abstracts of the articles were screened for inclusion by all authors, with the remaining articles reviewed in full text and the exclusion criteria applied. In the case of disparities between the authors’ judgments regarding suitability, they consulted to achieve agreement. Data from the included studies were extracted using a standard table developed specifically for this review (refer to Appendix A). The articles were summarised in terms of intervention type and participants, study aim and design, equipment, methods and main study results.

## RESULTS

The initial database and reference list searches conducted during November and December 2016 yielded a total of 120 unique articles. During the initial screening, 68 articles were excluded on title and another 33 articles were eliminated on abstract. The remaining 19 articles were reviewed in full-text. The full-length review excluded a further 12 articles, because they: (1) did not describe speech and language intervention via telehealth with the majority of participants between 4 and 12 years of age, and/or (2) did not provide outcome data on intervention effectiveness. From this selection process, seven articles were retained for the final systematic review. The review process is detailed in the flow chart in Figure 1.

### STUDY DESIGN

The seven included studies focused on telehealth-delivered speech and language intervention with primary school-age children. Two of the included studies were randomised controlled trials (level of evidence II) (Grogan-Johnson, Alvares, Rowan, & Creaghead, 2010; Grogan-Johnson et al., 2013) and another two studies were method comparison studies (level of evidence IIIa) (Gabel, Grogan-Johnson, Alvares, Bechstein, & Taylor, 2013; Grogan-Johnson et al., 2011) which were investigating the validity of telehealth-delivered intervention by comparing it with in-person results. A further three studies used a pre versus post study design (level of evidence IV) to determine if telehealth-delivered intervention facilitated improvement in the participants’ communication skills, with no comparison group (Fairweather, Lincoln, & Ramsden, 2016; Isaki & Farrell, 2015; Jessiman, 2003). Four of these papers included a participant satisfaction survey (Fairweather et al., 2016; Grogan-Johnson et al., 2010; Isaki & Farrell, 2015; Jessiman, 2003). Only one of the seven studies was conducted in Australia (Fairweather et al., 2016). The intervention services provided within the included studies were undertaken within a structured school/university clinic (n=6) or community health clinic (n=1) environment. The studies varied according to the intervention focus and outcome measures used.

### PARTICIPANTS

The majority of the studies (71%) focused only on primary school-age children between the ages of 4 and 12 years (Fairweather et al., 2016; Gabel et al., 2013; Grogan-Johnson et al., 2010; Grogan-Johnson et al., 2011; Grogan-Johnson et al., 2013; Isaki & Farrell, 2015; Jessiman, 2003). However, one study included a very small number of participants from 3 years of age (exact number of participants not specified) (Fairweather et al., 2016) and another included one participant aged 15 years (Gabel et al., 2013). Although these few participants were aged outside of the set criteria, the majority of the participants in the studies were aged between 4 and 12 years, allowing the results to be suitably applied to the primary school-age population. Other studies however were excluded during the initial study selection process due to the majority of participants being aged outside of the set criteria. Five of the seven studies (Fairweather et al., 2016; Grogan-Johnson et al., 2011; Grogan-Johnson et al., 2013; Isaki & Farrell, 2015; Jessiman, 2003) had small sample sizes (2 to 19) and the remainder had moderate sample sizes ranging from 38 to 71 participants.

### TELEHEALTH EQUIPMENT

Three papers reported the use of commercial videoconferencing systems (Gabel et al., 2013; Grogan-Johnson et al., 2011; Grogan-Johnson et al., 2013) designed for use with low-speed connections (using a 128 kbit/s internet link). In contrast, three studies reported the use of web-based videoconferencing platforms (Fairweather et al., 2016; Grogan-Johnson et al., 2010; Isaki & Farrell, 2015) and the final study used a custom telehealth videoconferencing system (Jessiman, 2003). Two studies complemented their telehealth equipment with document cameras (Grogan-Johnson et al., 2010; Jessiman, 2003). The seven reviewed studies used real-time videoconferencing.

### INTERVENTION TYPE, INTENSITY AND TARGETS

Five of the seven included studies investigated the application of both speech sound and language intervention through telehealth (Fairweather et al., 2016; Gabel et al., 2013; Grogan-Johnson et al., 2010; Isaki & Farrell, 2015; Jessiman, 2003). The remaining two studies focused primarily on the investigation of speech sound intervention (Grogan-Johnson et al., 2011; Grogan-Johnson et al., 2013). Notably, no studies included in the review solely examined the provision of language intervention through telehealth.

#### SPEECH SOUND INTERVENTION

Two studies conducted only traditional speech sound intervention (Van Riper approach to articulation intervention) through both telehealth and in-person delivery models. The participants in the study conducted by Grogan-Johnson et al. (2011) received 20 minutes of therapy each week between fall (baseline) and spring (post-intervention), whereas Grogan-Johnson et al. (2013) provided intervention for 30 minutes twice per week for a five week period. Both studies followed the same session format, however, only one study required the participants to reach a set number of productions prior to progressing through the intervention levels (Grogan-Johnson et al., 2013). The intervention targets in both studies were selected based on the participant’s current Individualised Education Plan (IEP) goals, with the Grogan-Johnson et al. (2013) study also selecting additional targets based on the results of pre-testing on the Goldman Fristoe Test of Articulation – second edition (GFTA-2) (Goldman & Fristoe, 2002).

#### COMBINED SPEECH AND LANGUAGE INTERVENTION

An examination of a combination of speech sound and language interventions was conducted in five of the seven studies. The duration of intervention varied between studies. Participants in the study by Fairweather et al. (2016) received six 30 minute sessions on a fortnightly basis over a 12 week period, whilst Jessiman (2003) provided hourly treatment sessions twice a week for two months and Isaki and Farrell (2015) provided weekly therapy for two blocks of 15 weeks. Grogan-Johnson et al. (2010) provided one group of participants with telehealth treatment for four months followed by in-person intervention for another four months, while the second group received in-person intervention for four months and then subsequently telehealth-delivered intervention for four months. Further detail regarding the number and frequency of sessions in this study was not provided. Gabel et al. (2013) provided intervention to the telehealth group for 20 minutes per week for one academic year.

Further differences between the studies focussing on both speech and language intervention related to whether or not the treatment sessions were provided on an individual basis or in a group setting. An individual format was adopted in three of the studies (Fairweather et al., 2016; Isaki & Farrell, 2015; Jessiman, 2003), however, in the remaining two studies, the participants in the telehealth groups received mainly individual therapy sessions with some small group sessions also conducted (Gabel et al., 2013; Grogan-Johnson et al., 2010). The in-person participants in these two studies received primarily group sessions with 2–4 students, with some students alternatively receiving an individual pull-out model of intervention (Gabel et al., 2013; Grogan-Johnson et al., 2010).

The intervention provided varied depending on the selected targets. Two studies selected intervention targets based on the participant’s IEP goals and objectives (Gabel et al., 2013; Grogan-Johnson et al., 2010), whereas another two studies established therapy goals based on recent assessment results (Isaki & Farrell, 2015; Jessiman, 2003). The fifth study developed goals in collaboration with adults familiar with each participant (Fairweather et al., 2016).

### OUTCOME MEASURES

The included studies examined the efficacy of telehealth intervention using various outcome measures. Six different outcome measures were investigated: the Goldman Fristoe Test of Articulation – second edition (GFTA-2); Functional Communication Measures (FCMs); goal achievement; informal probes; comparison of pre-intervention baselines with post-intervention production levels; and change reported on quarterly progress reports.

### EFFICACY OF THERAPY

#### GOLDMAN FRISTOE TEST OF ARTICULATION – SECOND EDITION (GFTA-2)

Three studies utilised pre- and post-intervention testing with the GFTA-2 to compare telehealth to in-person delivered intervention (Grogan-Johnson et al., 2010; Grogan-Johnson et al., 2011; Grogan-Johnson et al., 2013). Each of these studies revealed no significant difference between the two treatment modalities, with the first study reporting across three measurement points (pre-test p=0.16; post-first treatment period p=0.06; post-second treatment period p=0.21) and the second and third study reporting across two measurement points each (pre-test p=0.805; post-test p=0.805; and pre-test p=0.706; post-test p=0.644, respectively). Using a repeated measure ANOVA, Grogan-Johnson et al. (2013) found no significant difference between the two groups on post-intervention GFTA-2 testing (p=0.415); however, a statistically significant change in test scores was evident from pre- to post-intervention for both groups (p=0.020), indicating that both groups made significant and similar progress during intervention. Grogan-Johnson et al. (2011) identified a similar result with both groups making significant improvement in performance (p=0.014) but neither group was found to improve more than the other.

#### FUNCTIONAL COMMUNICATION MEASURES (FCMS)

Two studies measured outcomes through Functional Communication Measures (FCMs), which are used as a measure of progress in the ASHA K-12 Schools National Outcomes Measurement System (NOMS) database (American Speech-Language-Hearing Association, 2003; Gabel et al., 2013; Grogan-Johnson et al., 2010). This database reports descriptive information on students receiving in-person speech-language intervention in the school system. Gabel et al. (2013) compared their results for the telehealth condition with the subjects reported in the NOMS database (in-person participants). This study revealed similarities between the changes in FCM level for the telehealth group and also the in-person participants for disorders related to intelligibility (66.7% improved at least one level in telehealth and 62.3% in-person) and speech sound production (84.6% in telehealth and 78.4% in-person) (Gabel et al., 2013). For spoken language production, this study revealed a sizable difference between telehealth and in-person results, with 55.6% and 71.1% improving at least one level respectively. Gabel et al. (2013)’s results for spoken language comprehension were varied, with a higher percentage of telehealth participants improving by one level (47.1% vs. 38.2%) and a lower percentage improving by multiple levels in comparison to the in-person group (11.8% vs. 27.8%). The results reported by Gabel et al. (2013) were in contradiction to the results identified by Grogan-Johnson et al. (2010). As part of this research, the FCMs were used to compare progress between two groups of students, one group that received telehealth-delivered intervention and the other via in-person. This study found that a slightly lower percentage of participants in the telehealth group improved at least one level compared to the in-person group for disorders related to intelligibility (63% vs. 70%, respectively). This was similar for the speech sound production measure, with less participants in the telehealth group improving at least one level (71% telehealth vs. 79% in-person). However, for disorders related to spoken language production, a higher percentage of telehealth participants improved by a minimum of one level in comparison to the in-person group (72% vs. 62%, respectively).

The results of these two studies demonstrate conflicting findings; however, neither of the studies conducted statistical analyses of the results and thus the significance of the percentage differences between the two intervention conditions is unknown. The limitations evident in both studies could also likely have introduced confounding factors, which may have affected the results. For instance, one study had a considerable difference in the sample size for the two conditions and did not randomly allocate participants, but instead selected the telehealth participants from a pilot project already being conducted (Gabel et al., 2013). The selected participants were allocated to the telehealth condition and their results were compared with data already stored in the NOMS database, therefore introducing potential bias. Neither of the studies controlled for the type of service utilised (e.g., individual or group therapy) or the methods of treatment provided (Gabel et al., 2013; Grogan-Johnson et al., 2010).

#### GOAL ACHIEVEMENT

Two studies (Fairweather et al., 2016; Isaki & Farrell, 2015) used goal achievement to determine outcomes, with one study using Goal Attainment Scaling (GAS), a criterion-referenced measure of change rated on a five-point scale, to evaluate the telehealth program (Fairweather et al., 2016). This study revealed that 68.9% of the established goals were achieved at either an expected or greater than expected level. From the 19 participants, 15 (78.9%) achieved at least one goal at or above the expected level and eight participants (42.1%) achieved all their goals. The GAS scores were converted to t-scores to reflect performance above or below the expected level (e.g., achieving the set goal). This analysis revealed that 73.68% of the participants achieved or exceeded their set goal following six telehealth sessions.

The second study evaluated goal completion against a set criterion (Isaki & Farrell, 2015), with the results indicating that for the speech goals targeted, three of the five participants achieved 100%, one achieved 50% and the other achieved 33%. The three participants with language goals all achieved 100%. These results related only to telehealth and did not provide a comparison to in-person treatment.

#### INFORMAL PROBES

Examination of progress using informal probes was conducted in one study (Jessiman, 2003). The participants’ goals or number of goals were not detailed, however, based on informal probes completed after therapy and by parent report it was suggested that the participants made progress in their speech and language goals across the 12 sessions. One participant was reported to have made “substantial” progress while the other participant’s progress was “less substantial, but still appeared promising” (Jessiman, 2003, p.48–49). Jessiman (2003) quantified the participant’s progress using these terms by determining the number of speech and language skills mastered or progressing within the treatment period.

#### COMPARISON OF PRE-INTERVENTION BASELINES WITH POST-INTERVENTION PRODUCTION LEVELS

Two studies used the comparison of pre-intervention baselines with post-intervention production levels as an outcome measure (Grogan-Johnson et al., 2011; Grogan-Johnson et al., 2013). The analysis of this outcome measure differed between the two studies, however both studies indicated that progress was achieved regardless of the treatment modality. Grogan-Johnson et al. (2011) measured the change in speech sound production from baseline to the completion of intervention, with the results suggesting that both the telehealth (n=55) and in-person (n=8) groups made similar amounts of progress. The results were comparable for the percentage of improved baselines, with 98% (n=54) in the telehealth and 95% (n=6) in the in-person group. However, the in-person group had a higher percentage of unchanged baselines (2% [n=1] for telehealth and 12.5% [n=1] for in-person) and decreased baselines (0% [n=0] for telehealth and 12.5% [n=1] in-person) (Grogan-Johnson et al., 2011). The varying number of baselines targeted in the intervention may explain the difference in the results for unchanged and decreased baselines between the telehealth and in-person methods in this study. There were 55 baselines targeted for participants in the telehealth group and only eight collected for the in-person participants. Both groups only had one unchanged baseline however, due to the high variance in total baselines targeted, a considerable difference in percentage was indicated.

Grogan-Johnson et al. (2013) used listener judgments to compare pre- and post-intervention productions and these results were examined using a repeated measures ANOVA. The listener judgments revealed a statistically significant difference across time for both groups (p=0.007), but no significant difference between the two groups in regard to the amount of change across time (p=0.434). Thus, both groups were deemed to receive benefit from the intervention regardless of the service delivery model.

#### QUARTERLY PROGRESS REPORTS

The results reported on participants’ quarterly progress reports were used as outcome measures for two studies (Grogan-Johnson et al., 2010; Grogan-Johnson et al., 2011). In the study by Grogan-Johnson et al. (2010), quarterly student progress reports after the first treatment period identified that adequate progress or mastery was achieved for 75% (n=58 for telepractice and n=34 for in-person) of objectives in both conditions. A significant difference (p<0.05) between the two intervention conditions was indicated following the second treatment period, with mastery or adequate progress achieved for 88% (n=42) of objectives in the telehealth model and 84% (n=56) of objectives in the in-person model. A similar result was found in Grogan-Johnson et al. (2011)’s study, as more participants in the telehealth group (100%, n=25) mastered or made adequate progress on their IEP goals in comparison to the 87% (n=13) of participants in the in-person group.

The difference in the results for the number of IEP goals achieved between the intervention conditions in these studies can be explained by a disproportionate number of IEP objectives being targeted in the two intervention conditions and across the first (telehealth n=77, in-person n=45) and second treatment period (telehealth n=48, in-person n=67). A larger number of total IEP objectives were targeted in the telehealth group across the two treatment periods.

### PARTICIPANT SATISFACTION

Four studies reported satisfaction data through the provision of surveys (Fairweather et al., 2016; Grogan-Johnson et al., 2010; Isaki & Farrell, 2015; Jessiman, 2003). High levels of satisfaction with telehealth-delivered intervention and the progress achieved were found in all studies. Two studies reported that concerns were identified regarding the child’s reduced attention in telehealth sessions (Isaki & Farrell, 2015) as well as the need to improve internet connectivity, audio output and communication with stakeholders (Fairweather et al., 2016).

## DISCUSSION

The present review investigated the efficacy of telehealth-delivered SLP services when compared to traditional in-person delivery for primary school-age children with speech and/or language difficulties. Evidence was collated through a systematic review of the available telehealth literature. Overall, the findings of the review showed that there is some evidence to support the use of telehealth when delivering SLP intervention services to school-age children. However, it also demonstrated that the amount of research into speech and language intervention for children via the telehealth service delivery model is limited and of variable quality, as the included studies span across the levels of evidence according to the National Health and Medical Research Council (NHMRC) (Australian Government: National Health and Medical Research Council, 2009).

A total of six different types of outcome measures were used to investigate the efficacy of telehealth intervention, therefore creating difficulty in directly comparing the studies. The design of the three studies using the goal achievement and informal probe outcome measures did not allow direct comparison between the telehealth and in-person intervention conditions, as the study designs only evaluated the telehealth-delivered intervention, without comparing it to the traditional in-person model (Fairweather et al., 2016; Isaki & Farrell, 2015; Jessiman, 2003). However, these measures demonstrated considerable progress based on the targeted goals during the telehealth intervention.

The remaining reviewed studies directly compared the telehealth and in-person intervention conditions using four different outcome measures. There was convincing evidence in the literature suggesting that speech sound intervention delivered through telehealth to primary school-age children was just as effective as in-person intervention when measured through the GFTA-2 (Grogan-Johnson et al., 2010; Grogan-Johnson et al., 2011; Grogan-Johnson et al., 2013). Participants in both intervention conditions made significant improvements in performance and equal gains were demonstrated on the post-intervention testing.

Positive results were also identified in the studies that used the comparison of pre-intervention baselines and post-intervention production levels to measure outcomes, with both studies indicating that progress was achieved regardless of the treatment modality (Grogan-Johnson et al., 2011; Grogan-Johnson et al., 2013).

Despite these positive results, the two studies that examined telehealth-delivered speech and language intervention and used the FCMs as their outcome measure identified contradictory results. For the intelligibility and speech sound production measures, Gabel et al. (2013) found that more participants improved in the telehealth condition whereas, in the study by Grogan-Johnson et al. (2010), a lower percentage of participants improved in the telehealth condition when compared to the in-person condition, using the same measures. For the spoken language production measure, Gabel et al. (2010) found that a much lower percentage of participants improved in the telehealth condition compared to the in-person condition; however, Grogan-Johnson et al. (2010) found that more participants improved in the telehealth condition, again using the same measure. The authors did not conduct statistical analyses of these results, making it difficult to determine if the difference between the results is significant.

Both speech sound and language interventions were implemented as part of the seven studies included in the review. However, of these reviewed studies, there appeared to be a stronger focus on speech sound intervention, with two studies primarily aiming to assess this range of practice area through telehealth. The remaining five studies investigated the application of both speech sound and language intervention through telehealth, however two focused more heavily on speech than on language, as a greater number of speech goals were targeted in one study (Isaki & Farrell, 2015) and more speech-based FCMs were used as an outcome measure in another study (Grogan-Johnson et al., 2010). Overall, whilst the studies revealed that intervention delivered through telehealth is as effective as in-person intervention, this result seemed to be found more consistently with the provision of speech sound intervention than with language intervention. Although this suggests that speech sound intervention may be more suited to a telehealth approach, this finding is likely to be skewed by the more predominant focus on this range of practice area in the reviewed studies. Another possible explanation for this result is the difficulty in identifying comprehensive measures of language to be used when conducting research relating to telehealth-delivered services, as language is such a broad and highly variable range of practice area.

The uptake of the use of telehealth by speech-language pathologists has been influenced by the need to address the inequity of access to services experienced by Australia’s rural population (SPA, 2014). Telehealth allows services to be delivered to clients, including children, within their home and with the assistance of parents/carers, regardless of their location. The majority of the studies included in this review were however undertaken within a structured school or clinic environment, with little or no parent involvement. This results in difficulty drawing conclusions about the effectiveness of telehealth when implemented in the home setting, where the environment is likely to be less structured and full parent involvement is required.

Interestingly, all of the reviewed studies utilised real-time videoconferencing facilities, allowing the clinician and client to visualise each other. This finding is consistent with results from previous reviews (Mashima & Doarn, 2008; Reynolds et al., 2009; Taylor, Armfield, Dodrill, & Smith, 2014), indicating that real-time interactions support the delivery of services and strongly influence the clinical outcomes achieved through telehealth. Delivering speech and language intervention services through real-time videoconferencing facilities is an effective method of service delivery as this medium most closely resembles in-person interactions through the transmission of auditory and visual signals at a distance (Mashima & Doarn, 2008; Reynolds et al., 2009). SLP practice primarily consists of auditory, verbal and visual interactions, therefore allowing services to be easily translated into technology-based environments (Theodoros, 2012). This level of connection enhances the sense of clinician presence and facilitates the development of rapport between clinicians, clients and their families, provided that the necessary bandwidth is available to support the process (Mashima & Doarn, 2008; Reynolds et al., 2009).

Reports that videoconferencing facilities do effectively support real-time interactions between clinicians, clients and families, are consistent with parent, student, and staff satisfaction data that was collected as part of four of the seven studies included in this review (Fairweather et al., 2016; Grogan-Johnson et al., 2010; Isaki & Farrell, 2015; Jessiman, 2003). Satisfaction ratings were high across all stakeholders surveyed despite the concerns raised regarding slightly reduced attention by children in telehealth sessions and difficulties with technology. Similar findings with stakeholder satisfaction have been reported in various studies and reviews (Constantinescu et al., 2014; Crutchley & Campbell, 2010; Lincoln, Hines, Fairweather, Ramsden, & Martinovich, 2015; Mashima & Doarn, 2008; Sicotte, Lehoux, Fortier-Blanc, & Leblanc, 2003), indicating that relevant stakeholders deem telehealth as an effective method of delivering speech and language intervention to children. It is however, important to note that satisfaction ratings related to telehealth are likely to be particularly high in rural areas where in-person SLP services are not typically available.

Overall, the findings from the seven reviewed studies revealed that telehealth is a promising method for treating children with speech and/or language difficulties. However, in spite of this interesting finding, a number of methodological issues limit the quality of the results. The conclusions found in the literature on the effectiveness of telehealth-delivered intervention are dependent on the selected outcome measure. Outcomes for telehealth were more consistently positive when standardised assessments, such as the GFTA-2, were used for the pre- and post-intervention testing. The literature also revealed considerable variation in the intensity of therapy, with some studies claiming significant improvement after only a small number of sessions (6) were delivered fortnightly (Fairweather et al., 2016), whereas others reported on a larger number of sessions (10–12) that were delivered twice weekly (Grogan-Johnson et al., 2013; Jessiman, 2003), making the intervention format more intense. Additionally, the majority of the studies reviewed were based on a small and unequal sample size, resulting in difficulty generalising the results. Furthermore, of the four studies comparing the service delivery models, two studies did not randomly allocate participants to the intervention conditions, therefore introducing potential intervention condition bias. These differences in the studies made direct comparison difficult and therefore, may limit the weight of the findings. Thus, to provide further evidence regarding the effectiveness of telehealth-delivered intervention, studies that use more rigorous methods, such as randomisation of participants and power calculations, need to be performed to ensure that potential key findings can be accurately identified.

The current systematic review also has some limitations that require consideration. Firstly, although two studies included a very small number of participants outside the set age criteria, the primary school-age population was the focus of the review. Therefore, studies which included a large number of children outside this age range were excluded due to the differences in attention span and behaviour between age groups (Owens, 2012). Whilst this allows the results of the review to be appropriately applied to the primary school-age population, without the data being skewed from a mix of different populations, further research in this area is required to confirm if telehealth is as effective as in-person intervention when delivered in an early intervention format or to adolescents. Furthermore, the majority of the studies included in the review were undertaken in the USA, thus generalisability of the results to rural and remote communities within Australia is limited, due to factors such as the frequent lack of adequate and reliable internet connectivity (Australian Bureau of Statistics, 2016; Erdiaw-Kwasie & Alam, 2016; Park et al., 2015). Therefore, rural Australian communities may have difficulty supporting the telehealth service delivery model, an issue that may not have been adequately captured in this review.

## CONCLUSIONS

The current review aimed to determine if telehealth-delivered SLP interventions are as effective as traditional in-person delivery for primary school-age children with speech and language difficulties. The reviewed research was limited and of variable quality, however, the evidence presented showed that telehealth is a promising service delivery method for delivering speech and language intervention services to this population. This alternative service delivery model has the potential to improve access to SLP services for children living in geographically remote areas, reducing travel time and alleviating the detrimental effects of communication difficulties on education, social participation and employment. Although some initial positive findings have been published, there is a need for further research using more rigorous study designs to further investigate the efficacy of telehealth-delivered speech and language intervention.

## APPENDIX A

**Table 1:** Characteristics of Studies Identified in the Systematic Review

Study	Intervention and Participants	Study Aim	Methods	Results/Outcome
Fairweather et al. 2016 Australia	Intervention targeting speech sounds, receptive/expressive language, pragmatics and phonological awareness. N=19, average age 7.8 years (range 3–12yrs). Four SLPs	To investigate the effectiveness, feasibility and acceptability of a SLP teletherapy (TH) program for children in rural and remote areas.	**Study Design:** Pre/post design, reporting on degree of progress in TH tx as noted by GAS results. **Equipment:** Webcam enabled laptops, desktop computers or iPads, 1 of 3 low-bandwidth VC platforms (Adobe, Facetime or Skype), headsets and microphones **Procedure:** GAS goals developed in collaboration with supporting adults in child’s local environment. Participants received 6× 30mins SLP teletherapy sessions on a fortnightly basis using Come N See (CNS) program over a 12-week period. Semi-structured interviews conducted with parents four weeks prior to the conclusion of the sessions.	31 goals (68.9%) were achieved at either an expected or greater than expected level. Of the 19 participants, 15 (78.9%) achieved at least one goal at the expected level or beyond. 8 children (42.1%) achieved all goals. T-scores revealed 73.68% of the participants achieved at or above the expected level after up to 6 30-minute teletherapy sessions. Parents felt telehealth intervention was feasible but engagement and acceptability would be improved with regular communication between stakeholders.
Gabel et al. 2013 USA	Speech & language Tx Children. Grade – K-12 *Telepractice group* N=71, 63.4%M/ 36.6%F. Age 5–15 yrs. *NOMS database group* - N=5332, 67%M and 33%F Three SLPs	To study the effectiveness of a telepractice SLP program for school-age children by comparing data from a student sample receiving telehealth intervention with data from direct, in-person services	**Study Design:** Method comparison study, reporting on level of progress based on FCM scores. **Equipment:** Polycom videoconferencing software, desktop computers, webcam with built-in microphone, headsets, 128kbit/s internet link. **Procedure:** Participants in TH-led condition were compared to data from direct, in-person services available from the ASHA K-12 National Outcomes Measurement System (NOMS) database. Outcome data measured through FCMs. Participants in telepractice group received 20 minutes of therapy weekly.	70% of telepractice participants progressed one or more levels of the FCMs. Improvement varied across difficulties studied, but best outcomes identified for intelligibility and speech sound production intervention. Data compared favourably with NOMs database for same intervention. Data from telepractice participants receiving spoken language comprehension and production information differed from NOMs database with a higher percentage of participants making no progress and a lower percentage progressing multiple levels.
Grogan-Johnson et al. 2010 USA	Intervention for spoken language production, speech sound production and/or intelligibility. N=38 (13F, 25M). Age range 4–12 years. *Group 1* - N= 17 *Group 2* - N = 17 Four SLPs	To investigate the results of speech language therapy provided through TH compared to in-person tx.	**Study Design:** Single subject time-series (A–B) repeated measures design, reporting comparison across measurements taken at three points in time (beginning, middle and end of project). **Equipment:** Computer-based videoconferencing, headphones and a document camera. **Procedure:** Participants were treated in two groups – group 1 received TH tx for 4 months and then subsequently in-person therapy for 4 months. Group 2 received in-person therapy for 4 months, then TH therapy for 4 months. Participants were randomly allocated to the groups. Outcome measures were student progress on GFTA-2 and NOMS database, participant satisfaction and any interruptions to service delivery.	No significant difference in GFTA-2 scores between participants in the two treatment groups at pre-test (p=0.16); following the first treatment period (p=0.06) and second treatment period (p=0.21). Student progress reports after the first tx period identified that adequate progress or mastery was achieved for 75% of objectives in both conditions. Following second tx period mastery or adequate progress was achieved for 88% of objectives in TH and 84% of objectives for the in-person model – significant difference (p=<0.05). All participants expressed a high satisfaction with the delivery of services, progress achieved, comparison with in-person intervention and general attitude towards TH.
Grogan-Johnson et al. 2011 USA	Speech sound disorder intervention N=13 (11M, 2F). Age=6–11yrs. All children with a speech sound disorder. *Telehealth group* – *N=7* *In-person group* – N=6 Two SLPs	To examine whether speech intervention using computer-based materials with school-age students via telehealth is comparable to services delivered via a in-person SLP.	**Study Design:** Method comparison study, reporting statistical difference between TH and in-person conditions. **Equipment:** Desktop computer, webcam with microphone and headset. custom TH system with real-time VC with 128kbit/s internet link and TinyEYE Speech Therapy software. **Procedure:** Both groups received traditional speech sound intervention for 20 minutes weekly. Multiple measures of progress assessed: 1) Pre- and post-testing using GFTA-2; 2) comparison of pre-intervention baselines with production levels post-intervention; and, 3) comparison of quarterly progress reports.	No significant difference between the TH and in-person groups on the pre- (p = 0.805) and post-tests (p = 0.805). Both groups had a significant improvement in performance (p = 0.14). Children in both SDMs improved significantly in their speech production with the telehealth students demonstrating greater IEP goal mastery.
Grogan-Johnson et al. 2013 USA	Speech sound therapy N=14. *Telepractice group:* N=7, Avg age=8.4yrs, range= 6.4–9.9yrs *Side-by-side group:* N=7, avg age=9yrs, range= 7.9–10yrs Two SLPs	To investigate telehealth-delivered intervention services by comparing speech sound intervention delivered to children in either a telepractice or in-person delivery model in an intervention program.	**Study Design:** Method comparison study, reporting statistical difference between TH and in-person conditions. **Equipment:** Laptop, web-camera with microphone and headset. Polycom VC system with 128kbit/s internet link. **Procedure:** Both groups received traditional speech sound intervention for 30 minutes twice per week for a 5-week period. Participants were randomly assigned to either the in-person or TH condition. Multiple measures of progress assessed: 1) pre- and post-intervention testing conducted using subtests of GFTA-2; and 2) pre-and post-recording of single word identification task.	No significant difference found between two groups on post-intervention GFTA-2 through repeated measures ANOVA (p=0.415). No statistically significant difference between the mean listener judgements for the two groups on the pre-test (p=0.160) but a statistically significant difference in mean listener judgements across time for both groups (p=0.007). Thus, both groups benefitted from intervention and that benefit was the same regardless of intervention condition.
Isaki et al. 2015 USA	Speech and/or language intervention *Child participants* – N=5. Mean age 7.1yrs (range 4.5–9.8 yrs) *Adult participants* –(not reported in review)	To evaluate the effectiveness of Apple iPads to deliver telepractice speech and/or language services.	**Study Design:** Pre/post design, reporting on degree of progress in TH tx as noted by achievement of goals. **Equipment:** Apple iPads with Facetime. **Procedure:** All participants received individual telepractice therapy for a total of 15 weeks per academic semester. Sessions were provided weekly for 30–45 minutes.	Participants met the majority of their therapy goals with the paediatric participants meeting at least 33% of the speech goals and 100% of the language goals. Satisfaction surveys revealed no significant change of opinions about telehealth following the intervention (p>0.05). Clinicians indicated the need to resolve technical problems with use of iPads.
Jessiman 2003 USA	Speech sound therapy and improving understanding and use of language forms (noun and verb forms, & linguistic concepts) N=2. School-aged (exact age unknown) One SLP	Field report providing preliminary information on the use of the TH technology in the provision of speech and language assessment and treatment services for 2 school-aged children.	**Study Design:** Pre/post design, reporting agreement between TH and in-person conditions for assessment and degree of progress in TH intervention as noted by clinical observations, informal probes and parent feedback. **Equipment:** custom TH system with real-time VC, document camera, room cameras and television monitors **Procedure:** Structured Photographic Articulation Test conducted through TH then in-person 3 days later. Language Ax (TOLD-P:3) conducted only in-person. Tx conducted twice weekly for a 2-month period through TH. Client satisfaction documented via surveys obtained post-treatment.	Inconsistency with detection of speech sound errors between TH and in-person model. Accuracy increased with use of lapel microphones creating increased agreement between conditions. Child A and Child B progressed in their speech and language goals over the 12 sessions. Child A’s progress more substantial than Child B. Reliability and validity not reported. Parents reported satisfaction with the telehealth service and the gains child made during therapy.

Note. Ax = Assessment; CAS = Childhood Apraxia of Speech; F = Female; FCM = Functional Communication Measures; GAS = Goal Attainment Scaling; GFTA-2 = Goldman-Fristoe Test of Articulation – 2^nd^ edition, IEP = Individual Education Plan; M = Male; Mx = Management; N = number; SDM = Service delivery model; SLP = Speech Language Pathology/ist; TH = Telehealth; tx = treatment; VC = videoconferencing.
